# Erebosis is a new type of cell death for tissue homeostasis in the *Drosophila* intestine

**DOI:** 10.1371/journal.pbio.3001614

**Published:** 2022-04-26

**Authors:** Andreas Bergmann

**Affiliations:** UMass Chan Medical School, Department of Molecular, Cell and Cancer Biology, Worcester, Massachusetts, United States of America

## Abstract

Although there are over a dozen types of cell death known, there is clearly more to discover in this field. In this issue of *PLOS Biology*, erebosis is identified as a new type of cell death involved in tissue homeostasis of the adult *Drosophila* intestine.

The adult *Drosophila* intestine, specifically the posterior midgut, has become an important model for stem cell research. The cell lineage is quite simple. An intestinal stem cell (ISC) divides asymmetrically to generate a new ISC and an enteroblast (EB). The EB differentiates either into an enterocyte (EC) or an enteroendocrine cell [1]. ECs are absorptive epithelial cells that make up most of the cells of the intestine. Old ECs are removed by cell death and replaced by new ECs due to ISC activity. The R4 region of the posterior midgut has the highest turnover rate and renews every 4 to 7 days [2]. It is commonly believed that old ECs are dying by apoptosis. However, this has never been convincingly demonstrated, and the published data are often inconsistent and contrary. For example, in 2 studies, apoptosis inhibition resulted in fewer ISC divisions [2,3], while in another study, it had no effect on ISC activity [4]. Genetic inactivation of critical apoptotic components (Reaper, Hid, Grim, the caspase Dronc, and the apoptosome component Dark) does also not affect ISC activity [4]. Apoptotic labeling does not reliably stain ECs. Only the use of very sensitive apoptotic assays made it possible to detect caspase activity in ECs [5–7]. However, that activity occurred transiently at a sublethal level and supposedly does not result in apoptosis. In short, the role of apoptosis for the homeostatic turnover of ECs remains unclear, and alternative mechanisms may exist.

In this issue of *PLOS Biology*, Ciesielski and colleagues provided evidence for a different form of cell death that accounts for EC cell death [4]. They termed this type of cell death “erebosis” (from the Greek έρɛβος, meaning “deep darkness”). Erebosis was discovered somewhat serendipitously when the authors labeled adult midguts with an antibody that recognizes angiotensin-converting enzyme (Ance). Ance protein is not present in all ECs, but rather revealed a patchy immunolabeling in the R1, R2, and R4 regions of the midgut. Interestingly, colabeling of Ance with a variety of cellular and indispensable proteins showed a complementary or inverse pattern, meaning that the labeling of these proteins is low or even absent in Ance-positive cells (Fig 1). These proteins are components of essential complexes in the cell such as the cytoskeleton, septate and adherence junctions, nuclear membrane as well as of organelles such as mitochondria, endoplasmic reticulum (ER), and Golgi (Fig 1A). Ance-positive ECs also change their appearance. They become flat and line up at the basal muscle layer of the gut. Their nucleus flattens, and DNA labeling is often weak or even absent. The gold standard of defining cell death morphology is ultrastructural analysis. Consistently, immuno-electron microscopy (immuno-EM) confirmed a reduced number of mitochondria in erebotic ECs and reduced cytoplasmic content (Fig 1B). In addition, microvilli, which are necessary for nutrient uptake, are significantly shortened in erebotic ECs (Fig 1B). Combined, these data suggest that erebotic ECs are metabolically shutting down.

**Fig 1:**
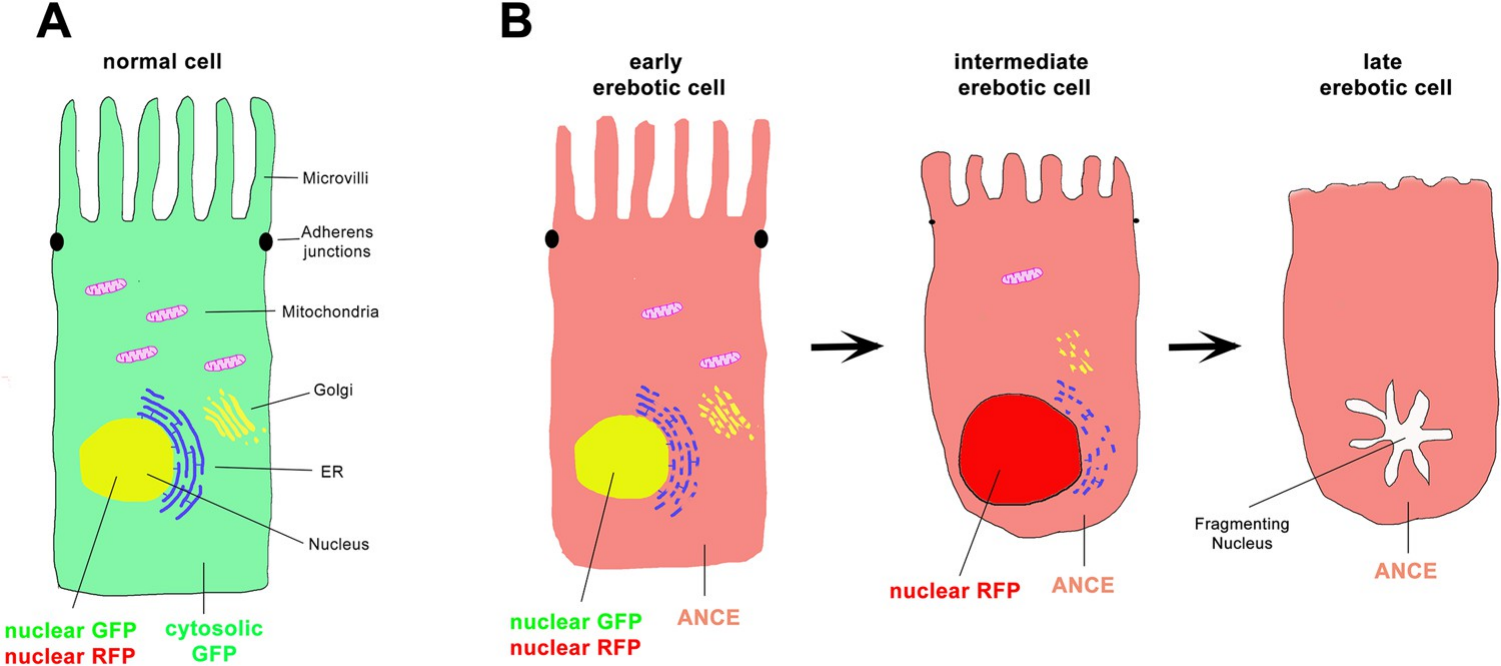
The morphology of erebosis. **(A)** Depicted is a normal EC expressing cytosolic GFP (illustrated in green) as well as nuclear GFP and RFP (illustrated in yellow). It also contains a nucleus, organelles (mitochondria, ER, and Golgi), cellular junctions, and microvilli. **(B)** Erebotic ECs are characterized by loss of proteins, organelles, and nuclear content as well as shorter microvilli. During early erebosis, cytosolic GFP is degraded, while nuclear GFP and RFP persists. Instead, erebotic ECs accumulate ANCE (illustrated in red in the erebotic cell)) through extracellular uptake. ANCE and GFP display a complementary staining pattern. At intermediate erebosis, nuclear GFP is degraded, but nuclear RFP (red) still persists. Erebotic nuclei are larger than normal, and loose nucleoli. At late erebosis, nuclear RFP is also degraded. Fragmented nuclei can be detected via TUNEL labeling. Microvilli are significantly shortened. ANCE, angiotensin-converting enzyme; EC, enterocyte; ER, endoplasmic reticulum.

Using transgenic proteins, GFP and nuclear RFP, the authors divided erebosis into 3 stages [4]. During early erebosis, cytosolic GFP disappears, while nuclear GFP and RFP remain (Fig 1B). At intermediate erebosis, all GFP signals are gone (Fig 1B), but nRFP still persists. At late erebosis, all GFP and RFP are lost (Fig 1B). Time-lapse imaging revealed that early erebosis occurs within 5 minutes. In contrast, intermediate and late erebosis together take about 12 hours. These observations raise the question, why does Ance accumulate in erebotic ECs, while all other proteins are reduced in abundance. In a surprising twist, the authors demonstrated that biosynthesis of Ance, a secreted protein, is actually not increased in erebotic ECs. In fact, extracellular Ance is taken up by erebotic ECs and accumulates there. Hence, Ance serves as a convenient marker for erebotic cells (Fig 1B). On the other hand, though, Ance is not involved in erebosis. Loss of function of *Ance* does not block erebosis, and overexpression of *Ance* does not trigger it.

Erebosis is not affected by mutants of apoptosis, autophagic cell death, or necrosis. Molecular markers of these death pathways also did not label erebotic cells with the exception of TUNEL, which labels DNA breaks in apoptotic cells. However, TUNEL labeling is only observed in a small fraction of erebotic cells, likely representing late erebotic cells, and RNA interference (RNAi) targeting caspase-activated DNase (CAD), which mediates TUNEL during apoptosis, does not block the TUNEL signal nor erebosis [4]. Another way by which ECs can be removed from the *Drosophila* midgut is through epithelial shedding (at least during bacterial infection) [8]. However, erebotic cells do not display any features of cell shedding. Combined, these data suggest that erebosis represents a distinct form of cell death.

Despite these exciting findings, a limitation of the work by Ciesielski and colleagues is the fact that they actually did not directly demonstrate that erebotic ECs are dying. They argue that the loss of essential proteins, DNA and organelles, is hardly compatible with normal cellular physiology [4], which is true, but the lack of convenient markers of erebosis (other than Ance) makes it difficult to trace these cells and determine their final fate. Very helpful in this matter would also be the identification of the genetic pathway involved in erebosis. Loss-of-function mutants of erebotic genes will allow to examine the phenotypic consequences of loss of erebosis and also reveal if erebosis occurs in other tissues and/or in areas of the intestine where Ance does not accumulate. Therefore, genetic identification of erebotic genes is an important task for future work.

There are many types of cell death. Two of them, apoptosis and autophagic cell death, are employed under normal physiological conditions. Necrosis is an uncontrolled type of cell death triggered by cellular insult, but there are also types of necrosis (necroptosis) that follow a coordinated genetic process. Other types of cell death (ferroptosis, pyroptosis, aponecrosis, paraptosis, entosis, NETosis, parthanatos, etc.) occur under nonphysiological or pathological conditions and are often associated with an inflammatory response. The discovery of a new, potential physiological cell death pathway, erebosis, represents an exciting advance for the cell death community. It will be critical in future work to identify specific markers of erebosis and the genes involved in this process. Finally, is erebosis conserved in mammals including humans, and are there any maladies associated with defective erebosis? While much work is needed, the answers to these questions will open exciting new research avenues.

## Abbreviations

ANCE: angiotensin-converting enzyme
CAD: caspase-activated DNase
EB: enteroblast
EC: enterocyte
ER: endoplasmic reticulum
immuno-EM: immuno-electron microscopy
ISC: intestinal stem cell
RNAi: RNA interference
